# The “Immune Rebellion” from the Intestines to the Liver: A Vicious Cycle That Causes the Liver to Collapse

**DOI:** 10.3390/metabo16020092

**Published:** 2026-01-25

**Authors:** Wan-Ting Wang, Jia-Le Tian, Shuo Gao, Mao-Bing Wang, Yang Luo, Xun Li

**Affiliations:** 1 The First School of Clinical Medicine, Lanzhou University, Lanzhou 730000, China; wtwang2021@lzu.edu.cn (W.-T.W.);; 2 Key Laboratory of Biotherapy and Regenerative Medicine of Gansu Province, Lanzhou 730000, China; 3 Department of General Surgery, The First Hospital of Lanzhou University, Lanzhou 730000, China; 4 Medical Engineering Center for Liver Repair and Regeneration of Gansu Province, Lanzhou 730000, China; 5 Hepatopancreatobiliary Surgery Institute of Gansu Province, Lanzhou 730000, China; 6 Clinical Research Center for General Surgery of Gansu Province, Lanzhou 730000, China; 7 Innovation Center for Advanced Particle and Nuclear Medicine Technologies, Lanzhou 730000, China

**Keywords:** gut–liver axis, gut immune microenvironment, immune dysregulation, gut barrier, therapeutic targets for liver disease

## Abstract

The gut immune microenvironment and the liver engage in intricate information exchange via the gut–liver axis. The disruption of these interactions plays a pivotal role in the formation and exacerbation of pathological damage to the liver. The gut immune microenvironment is not an independent layer of the gut barrier; rather, it permeates and regulates all other barrier functions, serving as the core coordinator. Disruption of the immune microenvironment in the gut–liver axis drives progression across the full disease spectrum—from steatosis to hepatitis, fibrosis, and even liver cancer—through the continuous influx of immune-stimulatory signals that overwhelm the liver’s intrinsic immune regulatory mechanisms. Dysfunction of innate immunity components, amplification of inflammatory factors and key cellular signaling pathways, activation of adaptive immune T cells, and systemic effects mediated by liver-derived inflammatory factors collectively form a disordered immune microenvironment. This damages the intestinal barrier and exacerbates liver disease via the gut–liver axis, leading to further intestinal injury, thus establishing a self-reinforcing vicious cycle. Current therapeutic strategies based on modulating the gut–liver axis microenvironment remain limited, yet studies have demonstrated that suppressing gut immune cells, cytokines, and signaling pathways can help delay liver disease progression. Hopefully, future combined, precise, and cutting-edge gut immunotherapies will provide more effective strategies for liver disease treatment.

## 1. Introduction

According to the 2010 Global Burden of Disease Project assessment, liver cirrhosis (LC) accounted for 2% of all deaths, as did hepatocellular carcinoma (HCC) and acute hepatitis, indicating that liver disease constitutes a substantial public health burden [1]. Between 2010 and 2019, the burden of liver disease caused by virus-related hepatitis gradually decreased due to the successful implementation of vaccination programs, aflatoxin reduction initiatives, and aggressive antiviral therapy. However, with improved living standards, increased obesity, and rising alcohol consumption, the incidence of liver disease driven by metabolic dysfunction-associated steatotic liver disease (MASLD) and alcoholic liver disease (ALD) has dramatically increased [2]. The spectrum of liver diseases is shifting from a predominance of viral hepatitis to a predominance of metabolic liver diseases. Over the past few decades, the global occurrence rate and prevalence of autoimmune liver diseases (AILDs) have shown an overall increasing trend, which is associated with improvements in clinical diagnostic capabilities and enhanced patient awareness of the disease [3]. Consequently, the “true” burden of AILDs may be severely underestimated. Effectively treating these conditions also represents a crucial component in alleviating the overall burden of liver disease. Current treatment modalities for these diseases primarily entail surgery, antifibrotic drugs, and immunosuppressants. However, their efficacy still faces fundamental challenges in treating the most prevalent metabolic liver diseases, autoimmune liver diseases, and advanced cirrhosis. Furthermore, there are obstacles in translating therapeutic innovations into public health practice [1]. Therefore, a new, promising direction is needed to revitalize the search for better liver disease treatments.

The unique anatomical, physiological, and immunological relationship between the liver and the gut has shifted liver disease treatment strategies from the traditional hepatocentric approach to a holistic view of the gut–liver axis. A substantial body of research demonstrates that dysregulation of the gut–liver axis serves as a central nexus in the pathogenesis and progression of various liver diseases. Consequently, numerous restorative interventions targeting gut barrier function have offered novel therapeutic avenues. For instance, targeting the gut microbiota (through probiotics, prebiotics, dietary interventions, or fecal microbiota transplantation) to restore gut barrier function offers promising new strategies for liver disease treatment [4,5]. The advantage of this approach lies in its intervention targeting the “upstream” origin of the disease, potentially offering broad-spectrum efficacy and lower side effects. However, therapeutic outcomes are subject to significant batch-to-batch variability, and the approach lacks precision, specificity and targeting capability. The intestinal immune system engages in dynamic crosstalk with the liver via the gut–liver axis. Following the disruption of intestinal immune tolerance, erroneous recognition and attack by the intestinal immune system lead to aberrant activation of the immune microenvironment and the formation of a pro-inflammatory microenvironment. This process generates numerous pro-inflammatory factors, which damage multiple intestinal barrier systems by, for example, disrupting epithelial tight junctions, inducing epithelial cell apoptosis, impairing the mucus layer, and altering the gut microbiota. These factors subsequently reach the liver via the bloodstream, impacting the hepatic ecosystem, thereby driving and sustaining liver inflammation, steatosis, and fibrosis, and ultimately affecting immune surveillance against hepatocellular carcinoma [6,7,8]. Immune system dysfunction serves as the critical bridge linking intestinal homeostasis imbalance to hepatic pathological injury. Therefore, targeting the intestinal immune microenvironment (e.g., by restoring intestinal immune tolerance or targeting specific immune cell subsets) offers a more stable and precise therapeutic strategy for treating various liver diseases.

Clearly, intricate information exchange occurs between the gut immune microenvironment and the liver via the gut–liver axis. Dysregulation of this communication plays a pivotal role in the formation and exacerbation of pathological damage to the liver. Therefore, we provide a review of the literature on the information exchange within the immune microenvironment via the gut–liver axis and its potential as a novel therapeutic target in liver diseases, aiming to offer new strategies for early intervention and the delay of liver disease progression.

## 2. The Role of the Gut Immune Microenvironment in the Pathophysiology of the Gut–Liver Axis

### 2.1. Anatomical and Physiological Basis of the Gut–Liver Axis

The gut–liver axis is a bidirectional communication system based on unique anatomical connections (the portal vein and biliary tract). Its physiological core lies in the circulation of bile acids, nutrients, and microbial metabolites, which mediate the continuous dialogue between the liver and the gut. It is widely recognized that the gut–liver axis rests on three major anatomical and physiological pillars [4,9]: (1) Portal venous system: Serving as the primary conduit, this system directly transports nutrients, microbial products, and metabolites absorbed from the intestine to the liver. (2) Biliary system: Bile acids, synthesized by the liver and secreted into the intestine via the bile ducts, participate in fat digestion and shape the gut microbiota. The microbiota, in turn, converts primary bile acids into secondary bile acids, some of which are reabsorbed by the liver (enterohepatic circulation). (3) Convergence of systemic circulation and mucosal immune system: The liver receives and processes factors derived from the intestinal immune microenvironment via the lymphatic and systemic circulation.

### 2.2. The Physiological Role of the Immune Microenvironment in the Multi-Tiered Defense System of the Gut Barrier

The gut barrier is a comprehensive functional unit composed of physical barriers (epithelial cells, tight junctions), chemical barriers (mucus layer, antimicrobial peptides), biological barriers (commensal microbiota), and immune barriers gut-associated lymphoid tissue (GALT), secretory IgA, lamina propria immune cells (e.g., macrophages, dendritic cells, T cells, B cells) [10,11]. The immune system is not an independent layer but rather a core coordinator that permeates and regulates all other barrier functions. The homeostasis of the immune microenvironment is crucial for maintaining overall barrier integrity and distinguishing between “friend” and “foe”. Its dysfunction is the root cause of various intestinal and extraintestinal diseases, such as inflammatory bowel disease and liver disease [10].

**Figure 1:**
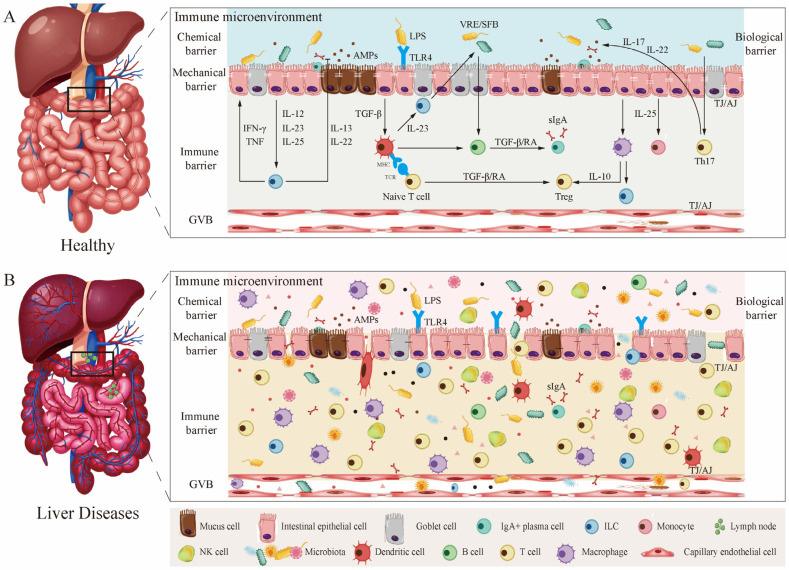
Gut immune microenvironment in the pathophysiology of the gut–liver axis. (**A**) Physiological homeostasis: A healthy intestinal barrier (intact epithelium, mucus layer, antimicrobial peptides, and secretory IgA) and immune tolerance in the lamina propria (Tregs, tolerogenic DCs) jointly maintain the stability of the gut–liver axis, preventing microbial translocation. (**B**) Pathological disruption: Disruption of the intestinal barrier (leaky gut) leads to the translocation of pathogen-associated molecular patterns (PAMPs) and damage-associated molecular patterns (DAMPs). Immune homeostasis in the lamina propria is disrupted, shifting towards a pro-inflammatory environment dominated by Th1/Th17 cells, M1-type macrophages, and pro-inflammatory cytokines (e.g., TNF-α, IL-1β), accompanied by reduced production of secretory IgA.

For instance, processes such as mucus secretion by goblet cells and antimicrobial peptide production by Paneth cells are strictly regulated through immune signaling, exemplified by cytokines like IL-22 and IL-13 [11]; Furthermore, goblet cells secrete mucus and specific proteins (e.g., RELMB) that actively transport luminal antigens to lamina propria dendritic cells via “antigen delivery” to induce immune tolerance. This process also modulates Th2 and Treg cell responses [12]. This clearly demonstrates that the chemical barrier function is finely regulated by the local immune microenvironment, while also actively shaping the immune response, serving as an indispensable component in achieving immune tolerance. Intestinal epithelial cells are far from being passive physical barriers; they constitute an active interface that directly communicates with immune cells through multiple intricate pathways. The immune microenvironment determines the “on–off” state of the barrier by regulating epithelial cell functions and tight junctions, while epithelial cells shape the nature of immune responses through signaling feedback, forming an inseparable bidirectional regulatory loop. The Gut Vascular Barrier (GVB) consists of specific cells and tight junctions in the intestinal capillary endothelium. Immune cells (e.g., resident intestinal macrophages) are crucial for maintaining tight junctions and barrier function in GVB endothelial cells through the secretion of WNT ligands [9]. This directly demonstrates the active role of the lamina propria immune microenvironment in maintaining anatomical barriers deep within tissues. Similarly, the immune microenvironment plays a crucial role in the resistance against colonization by intestinal pathogens. This protective role is regulated by the gut microbiota. For instance, microbial products such as lipopolysaccharide (LPS) and flagellin stimulate TLR4^+^ stromal cells and TLR5^+^CD103^+^ DCs, thereby promoting the expression of REGIIIγ, which inhibits the colonization of Gram-positive vancomycin-resistant enterococcus (VRE), in epithelial cells. Segmented filamentous bacteria (SFB), which adhere to the intestinal epithelium, enhance IgA production by B cells, SAA-dependent Th17 cell differentiation, the production of pro-inflammatory cytokines, and the generation of antimicrobial peptides in epithelial cells. These processes confer protection against citrobacter rodentium [13]. The microbiota and host immune system co-maintain an anti-inflammatory, tolerant, and defensively robust intestinal environment through reciprocal regulation via microbial metabolites. The breakdown of this homeostasis serves as a precursor event to barrier dysfunction (Figure 1A).

The intact gut barrier constitutes a multi-tiered fortress system wherein the functions of goblet cells, intestinal epithelial cells, the microbiota, and the GVB are highly dependent on signaling support from specific immune cells in the intestinal lamina propria. This provides the most direct molecular and cytological evidence for “immune microenvironment-mediated regulation of physiological barriers”.

### 2.3. Manifestations of Immune Microenvironment Disruption in the Gut–Liver Axis

The disruption of the intestinal immune microenvironment is primarily manifested in the following three aspects. First is the imbalanced ratio of pro-inflammatory to anti-inflammatory cytokines. For instance, secondary bile acids produced by gut microbiota metabolism can strongly inhibit the activation of the NLRP3 inflammasome in macrophages by activating the receptor TGR5, thereby reducing the maturation and release of the pro-inflammatory cytokines IL-1β and IL-18 [14]. An obesogenic diet can rapidly inhibit IL-22 production by innate lymphoid cells in the small intestine, leading to the suppression of STAT3 in intestinal epithelial cells (IECs) and the activation of the WNT-β-catenin signaling pathway. This causes the absorptive enterocyte compartment to expand, contributing to metabolic dysfunction-associated steatotic liver disease [15]. Second, immune cell subsets become imbalanced. Studies have shown that, compared with controls, mice with alcoholic hepatitis develop more severe liver inflammation, which is associated with increased intestinal permeability and higher bacterial translocation due to an increase in CD4^+^T and NKT lymphocytes in the mesenteric lymph nodes [16]. This dysregulation of the immune microenvironment in the intestinal lamina propria, in turn, impairs the barrier function of the intestinal epithelium, subsequently triggering liver inflammation. In liver disease, the number of Tregs in the gut and liver is reduced or their function is impaired, weakening their inhibitory effect on effector T cells (e.g., Th1, Th17). This leads to an imbalance between immunosuppressive and effector subsets, resulting in intestinal barrier damage and liver disease progression [17]. Finally, mucosal immune tolerance breaks down. For example, as mentioned earlier, the GVB serves as the final anatomical defense against bacterial translocation. Its integrity is maintained by the resident CX3CR1^+^ macrophage subset in the intestinal lamina propria through the secretion of WNT ligands [9]. Secretory IgA (sIgA) is the core executor of mucosal immune tolerance. It coats commensal bacteria through an immune exclusion mechanism, limiting their contact with and translocation across the epithelium without triggering strong inflammation. Alterations in the quality or quantity of sIgA can lead to dysbiotic localization of the microbiota and excessive epithelial contact, thereby breaking tolerance and activating local immune responses [18]. Functional defects in sIgA are an early and significant marker of immune microenvironment disruption in the gut–liver axis, directly contributing to gut microbiota dysbiosis and immune activation. Furthermore, recent studies indicate that in MASLD, viable bacteria can translocate to the mesenteric adipose tissue (MAT) and form local microabscesses, activating immune responses within the MAT. This creates a persistent extraintestinal inflammatory focus, in which inflammatory mediators are continuously released into the portal vein, exacerbating liver inflammation [19]. These ectopic, microbiota-driven immune activation foci become independent sources of inflammation that persistently drive liver injury, representing a novel paradigm of immune dysregulation in the gut–liver axis (Figure 1B).

The disruption of the immune microenvironment within the gut–liver axis is, in essence, a pathological shift in the liver from an immune-tolerant organ to a chronic inflammatory milieu, with the breakdown of intestinal immune homeostasis serving as the central driver of this transition.

## 3. Dynamic Crosstalk Between Liver and Gut: Immune Dysregulation–Intestinal Leakage–Gut–Liver Axis–Liver Disease Progression

**Figure 2:**
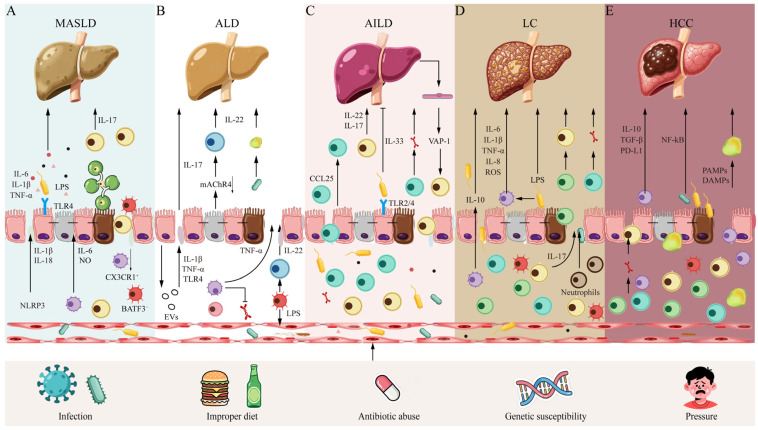
Dynamic mechanisms of gut–liver axis disruption driving chronic liver disease progression: From immune dysregulation to liver cancer.

Multiple external triggers disrupt immune tolerance within the gut–liver axis, impairing gut barrier function. This leads to overactivation of the immune system, which persistently activates the liver’s innate immune system via the gut–liver axis, thereby exacerbating the disruption of hepatic immune tolerance and establishing a vicious inflammation–injury cycle. This disruption of the intrahepatic immune microenvironment triggered by gut immune dysregulation constitutes a common core pathway driving the progression of multiple liver diseases, including MASLD, ALD, AILD, LC, and HCC (Figure 2).

Intestinal immune dysregulation and barrier disruption drive the progression of chronic liver disease to its end-stage through the gut–liver axis. (A) MASLD: Risk factors such as metabolic abnormalities disrupt intestinal homeostasis, and mild intestinal permeability triggers hepatic steatosis and chronic inflammation. (B) ALD: Persistent alcohol exposure exacerbates intestinal inflammation and barrier loss, driving significant hepatic inflammation. (C) AILD: In genetically susceptible individuals, intestinal permeability may lead to the translocation of self/mimic antigens, breaking immune tolerance and triggering autoimmune attacks against the bile ducts or hepatocytes. (D) LC: Severe gut–liver axis disturbance substantially increases the risk of antigen/bacterial translocation, accompanied by systemic inflammation and complications, forming a vicious cycle. (E) HCC: Against the backdrop of chronic inflammation and regeneration, the accumulation of genetic mutations, dysregulation of immune surveillance, and infiltration of tumor-associated macrophages and immunosuppressive cells collectively promote malignant transformation.

### 3.1. MASLD

MASLD has become the most prevalent chronic liver disease worldwide, with its disease spectrum progressing from simple hepatic steatosis to metabolic dysfunction-associated steatotic hepatitis (MASH), hepatic fibrosis, cirrhosis, and ultimately hepatocellular carcinoma. The traditional “second hit” theory has evolved into a more complex “multiple parallel hits” model, wherein dysfunction of the gut–liver axis–particularly dysregulation of its immune microenvironment–is recognized as the core driver initiating disease progression and the malignant transformation from benign steatosis to inflammatory MASH and fibrosis. The intensive immune and metabolic crosstalk between the gut and liver occurs via the portal vein, biliary tract, and systemic circulation. Disruption of homeostasis along this axis constitutes the pathophysiological cornerstone of MASLD (Table 1).

#### 3.1.1. Innate Immunity

Hepatic macrophages play a pivotal role in the progression of MASLD. Studies have demonstrated that intestinal CX3C chemokine receptor 1-positive (CX3CR1^+^) macrophages contribute to maintaining intestinal barrier stability and inhibiting gut microbiota translocation. Intestinal CX3CR1 knockout mice lack CX3CR1^+^ macrophages, leading to impaired function of the multi-layered intestinal barrier and accelerated progression of MASH via the gut–liver axis [29,30]. Furthermore, in patients with MASH, intestinal macrophages exhibit increased release of nitric oxide, which further inhibits the repair of the intestinal epithelial barrier mediated by connexin 43. Concurrently, intestinal macrophages release increased amounts of IL-6, which modulates the expression of the tight junction protein claudin-2 in intestinal epithelial cells, thereby affecting intestinal epithelial barrier function and accelerating the progression of MASLD [31]. These findings indicate that the population of intestinal pro-inflammatory macrophage subsets is increased and capable of secreting pro-inflammatory cytokines, leading to intestinal inflammation and intestinal barrier disruption, with a subsequent influence on the MASLD process via the gut–liver axis.

#### 3.1.2. Key Pathway Amplification

As previously mentioned, activation of the NLRP3 inflammasome pathway in the intestinal immune microenvironment generates large amounts of IL-1β and IL-18, which further damage the intestinal barrier and ultimately exacerbate MASLD [14]. The intestinal immune system recognizes pathogen/microbe-associated molecular patterns (P/MAMPs) via pattern recognition receptors such as TLRs and nucleotide-binding oligomerization domain (NOD)-like receptors (NLRs). Subsequently, the P/MAMPs influx resulting from pathological bacterial translocation (BT) and/or excessive increase in susceptibility induces the release of pro-inflammatory cytokines (e.g., TNF-α, IL-1, IL-6), chemoattractants, and eicosanoids from monocytes. Upon reaching the liver, these mediators drive the generation of a local pro-inflammatory hepatic microenvironment [7]. Furthermore, when the transcription factor basic leucine zipper ATF-like transcription factor 3 (BATF3), which is essential for the development of intestinal DCs in mice, is knocked out, the localization of tight junction proteins in intestinal epithelial cells is altered, intestinal permeability increases, the mice develop metabolic syndrome, and the progression of hepatic steatosis is promoted [32].

#### 3.1.3. Adaptive Immunity Is Involved

Gut-derived antigens may disrupt hepatic immune tolerance, activating CD8^+^ cytotoxic T cells and CD4^+^ helper T cells (such as Th1 and Th17), while simultaneously impairing Treg function. This shifts immune attacks toward hepatocytes. In mice fed a high-fat diet (HFD), the percentage ratio of Th1/Th2 cells and Th17/Treg cells was imbalanced in the mesenteric lymph nodes (MLNs), and MLN CD4^+^ T lymphocytes tended to migrate to the liver, promoting hepatic inflammation [33,34]. In mice with MASH, intestinal IL-17 levels are elevated; intestinal Th17 cells migrate to the liver and secrete IL-17, thereby activating monocytes, bile duct epithelial cells, hepatic KC, and hepatic stellate cells (HSCs). This activation further promotes the secretion of pro-inflammatory cytokines and chemoattractants, exacerbating liver inflammation [35]. Conversely, in HFD-fed mice, donor-specific intestinal Th17 cells, induced in an IL-17-dependent manner, were shown to modulate intestinal epithelial lipid absorption by regulating the expression of the intestinal fatty acid transporter CD36, thereby attenuating the development of metabolic syndrome [36].

#### 3.1.4. Systemic Effects

Inflammatory factors produced by the liver can further damage the intestinal barrier, forming a reverse liver–gut injury pathway. Concurrently, hepatic steatosis itself exacerbates oxidative and endoplasmic reticulum stress, acting synergistically with gut-derived inflammatory signals. Studies have reported an increased number of CD8^+^ T cells in the jejunum of obese patients. Certain soluble factors secreted by these cells significantly inhibit pSer473-Akt activity in intestinal epithelial cells, thereby impairing insulin sensitivity in small intestinal epithelial cells and affecting small intestinal function [26]. However, several other studies have found that in the duodenum of patients with MASLD and metabolic dysregulation, the expression of CD4^+^ and CD8^+^ T lymphocytes and ZO-1 is significantly reduced, leading to impaired intestinal mechanical barrier integrity [27,28]. This further contributes to the selective loss of intrahepatic CD4^+^ T lymphocytes in humans [37].

In summary, disruption of the gut–liver axis immune microenvironment serves as the driving force for the transition of MASLD from benign hepatic fat accumulation to inflammatory, progressive liver diseases (MASH and fibrosis). This is not merely a failure of a single component, but rather a collapse of the entire system involving gut ecology, physical barrier, immune signaling, metabolic regulation, and intrahepatic cellular network. (Figure 2A).

### 3.2. ALD

The cause of ALD progression is far from being as simple as the direct toxicity of alcohol to hepatocytes. Previous research has established that persistent disruption of the immune microenvironment in the gut–liver axis serves as the core mechanism driving the malignant transformation of the disease from simple steatosis to hepatitis and fibrosis. Alcohol acts as the initiating factor by first disrupting the gut ecology and immune balance, after which injury signals are amplified and transmitted to the liver through multiple pathways (Table 2).

Initiation of intestinal barrier disruption. Alcohol directly damages the intestinal epithelium or disrupts tight junctions by perturbing local intestinal immunity (e.g., increasing pro-inflammatory IL-17), leading to the translocation of endotoxins such as LPS. Chronic alcohol consumption suppresses the expression of the muscarinic acetylcholine receptor M4 (mAChR4) in small intestinal goblet cells, impairing intestinal immune surveillance. This results in reduced formation of goblet cell-associated antigen passages (GAPs) and inhibition of the ILC3-IL-22 antimicrobial pathway, thereby compromising antimicrobial immunity and facilitating bacterial translocation to the liver [44]. Studies indicate that chronic alcohol intake activates intestinal monocytes/macrophages, prompting them to release TNF-α. This subsequently activates the expression of tumor necrosis factor α receptor I on intestinal epithelial cells, further promoting the activation of myosin light-chain kinase in these cells. This cascade ultimately leads to the loss of tight junction proteins and increased intestinal permeability [45]. Recent research demonstrates that in ALD, inactivated postbiotics can activate intestinal innate immunity by stimulating the intestinal DCs-ILC3 axis, upregulating IL-22 production. This enhances the intestinal barrier upstream, reduces bacterial translocation, and exerts reparative effects in the liver via the gut–liver axis [46]. Alcohol-associated intestinal dysbiosis, which alters gut-derived immune signals, serves as a significant driver of dysfunction in intrahepatic innate immune cells (e.g., NK cells). This constitutes another pathway for immune crosstalk along the gut–liver axis, influencing hepatic immune surveillance and the inflammatory milieu [47].

Multifaceted signal transmission. Along with viable bacteria and their products (e.g., LPS), damage-signaling vesicles (EVs) released by intestinal epithelial cells and imbalanced microbial metabolites also translocate to the liver, collectively forming a “mixed arsenal” that attacks the liver. Interestingly, alcohol stimulation alters both the quantity and cargo of EVs released by intestinal epithelial cells, with an increase in pro-inflammatory cytokines (e.g., TLR4, TNF, IL-1β) and a decrease in ZO-1, leading to increased intestinal permeability. These vesicles reach the liver via the portal vein, where they can directly impair hepatocyte viability and promote lipid accumulation [48]. Alcohol-induced dysbiosis, characterized by an imbalance in the microbial community, also leads to a reduction in microbial metabolites known as indoles. This reduction contributes to decreased levels of IL-22, a cytokine essential for maintaining intestinal integrity. The decline in IL-22 weakens the intestinal barrier, promotes bacterial translocation to the liver, and advances the progression of ALD [49]. Recent studies have shown that in alcohol-fed mice, the number of intestinal conventional type 1 dendritic cells (cDC1s) is significantly reduced, which exacerbates the downregulation of antimicrobial peptides. Consequently, the intestinal barrier is weakened and becomes more permeable, allowing bacterial toxins such as LPS to enter the bloodstream, thereby increasing inflammation and liver injury [50]. A certain concentration of ethanol within the intestinal lumen can activate intestinal mast cells, leading to the release of substances like histamine, which in turn increases intestinal mucosal permeability. This process also contributes, to some extent, to accelerating the progression of liver disease [51].

Vicious cycle. Ethanol-fed mice and patients with alcohol use disorder exhibit reduced duodenal macrophage numbers, dysregulated cytokine secretion, impaired phagocytic function, and diminished intestinal IgA secretion. Furthermore, even in the pre-cirrhotic stage, patients demonstrate reduced intestinal T cell populations and immune dysfunction, further exacerbating the risk of gut-derived liver inflammation [52].

Alcohol disrupts gut immune surveillance and barrier function, leading to leakage of intestinal vesicles, cytokines, immune cells, bacteria, and their products into the liver. This activates hepatic immune cells, triggering severe inflammation and forming a vicious cycle of “gut immune dysregulation–intestinal leakage–liver injury”—a core mechanism exacerbating alcoholic liver disease (Figure 2B).

### 3.3. AILD (e.g., Primary Biliary Cholangitis, Autoimmune Hepatitis)

AILD is a group of diseases characterized by the loss of immune tolerance in the liver, the presence of autoantibodies, and chronic inflammation, whose exact etiology has not yet been fully elucidated. Over the past decade, advances in microbiome research have positioned the gut–liver axis theory as a novel perspective for understanding environmental triggers and systemic immune dysregulation in AILD (Table 3). Although primary biliary cholangitis (PBC), autoimmune hepatitis (AIH), and primary sclerosing cholangitis (PSC) have distinct targets, clinical manifestations, and classic pathological mechanisms, they share an increasingly recognized common background: immune microenvironmental dysregulation of the gut–liver axis. This section will elaborate on the central role of immune microenvironmental dysregulation within the gut–liver axis in the pathogenesis and progression of AILD from this shared perspective, focusing on three aspects: erroneous homing, molecular mimicry, and signal amplification.

#### 3.3.1. Aberrant Homing

The inflamed liver/abnormal bile ducts aberrantly express “postal codes” (e.g., CCL25, MAdCAM-1) that are normally exclusive to the intestine. This misdirects immune cells (CCR9^+^/α4β7^+^ T cells, IgA^+^ plasma cells) originally destined for intestinal duty into the hepatobiliary system, resulting in bystander injury [53,59]. Within the intestinal lymph nodes, these antigens drive the differentiation of naïve T cells into pro-inflammatory Th17 cells. These Th17 cells migrate to the liver, where they secrete cytokines such as IL-17 and IL-22, recruiting neutrophils and promoting hepatocyte damage and portal inflammation [55]. Intestinal Bifidobacterium modulates the IL-33-induced Treg/Th17 imbalance via the TLR2/4 signaling pathway, thereby alleviating autoimmune hepatitis [60].

#### 3.3.2. Molecular Mimicry

Certain components of the gut microbiota (e.g., bacterial enzymes) exhibit high similarity to human autoantigens (e.g., mitochondrial enzymes). Antibodies and T cells produced by the gut immune system to combat these bacteria may mistakenly recognize and attack bile duct cells expressing similar autoantigens, triggering autoimmunity [54]. Intestinal pIgA antibodies form immune complexes that deposit around bile ducts, causing bystander injury to bile ducts through the activation of the complement pathway [53].

#### 3.3.3. Signal Amplifier

Abnormal LSECs act as an accomplice by upregulating molecules such as VAP-1, firmly anchoring recruited gut immune cells in the liver and promoting their infiltration into lesion sites, thereby amplifying the damage [58].

Research over the past decade has firmly established the central role of the gut–liver axis in the pathogenesis of AILD. The gut serves not only as the source of immune disruption but also as the epicenter driving liver-specific autoimmune responses (Figure 2C).

### 3.4. LC

The onset and progression of LC extend beyond the liver itself and are profoundly influenced by the bidirectional vicious cycle of interaction within the gut–liver axis. From hepatic inflammatory changes to fibrosis and ultimately cirrhosis, the gut immune microenvironment progressively deteriorates, imposing an additional burden on liver function. The first stage involves the breach of immune sentinels, where increased gut permeability allows bacteria and their byproducts, fungi, and other pathogens to penetrate and assault the gut immune system. The second stage manifests as sluggish and dysfunctional innate immune responses. The third stage features the failure of adaptive immunity and the loss of tolerance, characterized by functional exhaustion and increased apoptosis of intestinal T and B lymphocytes. Ultimately, irreversible systemic alterations occur, where persistent translocation of gut-derived pathogens results in recurrent, low-grade bacteremia, triggering systemic inflammation (Table 4).

In acute-on-chronic liver failure (ACLF), the intestinal barrier is severely compromised, leading to the massive and persistent translocation of bacteria and their products (e.g., LPS). This sustained stimulation exhausts the phagocytic and clearance capacity of the hepatic and systemic monocyte–macrophage system and induces the production of high levels of the immunosuppressive factor IL-10 (which may be related to type I interferon signaling) [65]. The direct clinical consequence is that patients face an extremely high risk of bacterial infection, which is a primary factor triggering ACLF. Macrophage-derived inflammasomes triggered by bacterial translocation and tissue damage also contribute to inflammatory liver injury [66]. Activated inflammasomes initiate caspase-1-dependent production of pro-inflammatory cytokines such as IL-1β and IL-18, which subsequently exacerbate hepatic inflammation, fibrosis, and injury in conditions like ALD, MASH, and other forms of liver damage [67]. Studies have shown that in MASH, intestinal B cells can directly migrate to the liver and promote the activation of metabolic CD8^+^ T cells, driving autoimmune liver injury; they also directly facilitate the development of liver fibrosis via the IgA-Fc receptor signaling pathway [68]. Intestinal immune imbalance leads to decreased expression of tight junction proteins, increased intestinal permeability, and the entry of pro-inflammatory factors and PAMPs into the liver, thereby promoting the activation of hepatic stellate cells.

Gut-derived LPS can stimulate TLR4 on quiescent HSCs and regulate TGF-β in a MyD88–NF-κB-dependent manner to activate these cells, thereby inducing a pro-fibrotic transformation and accelerating liver fibrosis [69]. Neutrophils are recruited to the liver via adhesion molecules ICAM-1 and VCAM-1 expressed on LSECs. In cirrhosis, neutrophil function is also disrupted by the dysfunctional gut–liver axis, leading to inflammation-associated hepatocyte injury and IL-17-dependent HSC activation [70].

In summary, in LC, immune dysregulation of the gut–liver axis forms a self-reinforcing vicious cycle. Breaking this cycle requires a dual strategy that both enhances immune defense and suppresses local inflammation/fibrosis, which represents the primary challenge and direction for current therapeutic development (Figure 2D).

### 3.5. HCC

As summarized previously, alterations in the gut immune microenvironment constitute one of the critical factors driving the late-stage deterioration and qualitative changes in liver cirrhosis. During the HCC phase, the gut immune microenvironment not only persists in the immunodeficient and dysregulated state characteristic of the cirrhotic stage but is also actively reshaped by the tumor itself and its derived signaling molecules, forming a gut–liver axis immune microenvironment conducive to tumor growth and metastasis (Table 5).

Intestinal immunosuppression and functional exhaustion. Intestinal immunity exhibits functional exhaustion and suppressive polarization, including reduced secretory IgA, T-cell exhaustion (with high expression of PD-1/TIM-3), expansion of regulatory T cells, and macrophage skewing toward immunosuppressive phenotypes (e.g., M2/TAM) [71,72,73,75]. This leads to the collapse of the mucosal barrier, transforming it from a defensive frontline into an open channel that permits the passage of tumor-promoting signals.

The carcinogenic remodeling of gut microbiota. Characteristic gut dysbiosis (such as expansion of Enterobacteriaceae) continuously delivers substances such as endotoxins, driving chronic intrahepatic inflammation and the activation of pro-carcinogenic pathways, including NF-κB, thereby forming a persistent oncogenic signaling engine [72].

The tumor actively shapes an immunosuppressive environment, characterized by a malignant dialogue within the gut–liver axis. HCC actively influences intestinal immunity and microbiota through secreted factors and metabolic alterations; meanwhile, the dysregulated gut continuously delivers PAMPs/DAMPs and carcinogenic metabolites (e.g., DCA) to the liver via a leaky gut [71]. Substances such as DCA can suppress the function of intrahepatic NK/NKT cells and promote M2-type TAM polarization. This bidirectional “malignant crosstalk” results in massive accumulation of myeloid-derived suppressor cells, M2 macrophages, and regulatory T cells in the liver, forming a potent immunosuppressive network [74].

The above mechanisms do not exist in isolation but constitute a self-sustaining immunosuppressive circuit. The exhausted intestinal gateway opens a channel for oncogenic signals, which drive inflammation and assist the tumor in recruiting immunosuppressive cells; in turn, the tumor further exacerbates intestinal dysfunction. The temporal sequence of “cause and effect” within this circuit, as well as its dynamic changes across different molecular subtypes of HCC or different treatment stages, still lacks evidence from human studies. Moreover, the effects of microbial metabolites are pleiotropic and act in a concentration-dependent manner; their actual effector networks in humans are likely far more complex than currently understood (Figure 2E).

## 4. Therapeutic Targets for Liver Disease: Strategies Based on Enhancing Gut Immune Barrier Function

**Figure 3:**
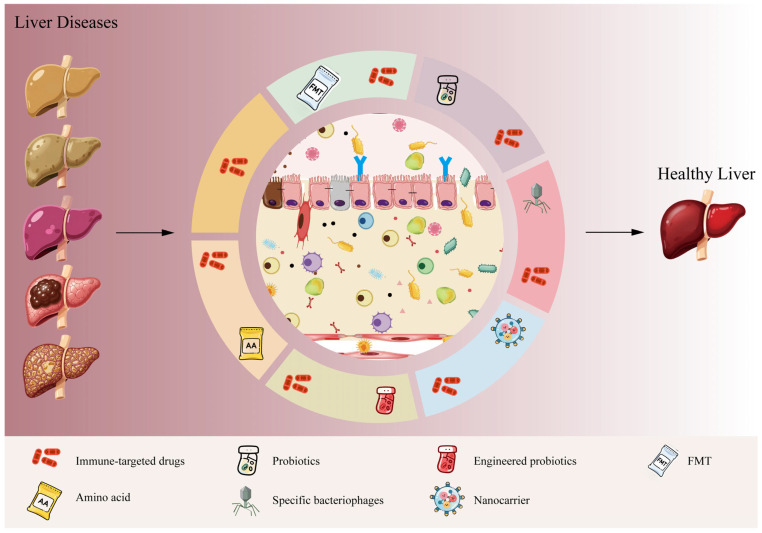
Targeting the gut immune barrier to improve the gut–liver axis: Innovative therapeutic strategies for liver diseases. This figure systematically summarizes novel therapeutic strategies for liver diseases aimed at breaking the vicious gut–liver axis cycle by improving gut immune barrier function; such approaches include immunotargeted monotherapy and comprehensive intervention strategies. Immunotargeted monotherapy directly modulates local gut immunity, suppresses excessive intestinal inflammation, and alleviates liver inflammation triggered by gut-derived antigens. The comprehensive intervention strategy enhances efficacy and precision by combining immunotargeted drugs with other methods. These strategies share the common goal of repairing the gut immune barrier, fundamentally reducing the translocation of pathogens and harmful substances to alleviate the liver’s immune burden, break the vicious inflammation–fibrosis cycle, and thereby delay or reverse liver disease progression.

Disruption of the gut immune microenvironment constitutes the pivotal initiating event that disrupts gut–liver axis homeostasis. This impairment of gut immune homeostasis serves as the core driver, overwhelming the liver’s intrinsic immune regulatory mechanisms through persistent delivery of immune-stimulatory signals. Consequently, it drives full-spectrum progression from steatosis to hepatitis, fibrosis, and ultimately liver cancer. This intrahepatic immune microenvironment disruption, triggered by gut immune dysregulation, represents the central common pathway underlying disease progression across multiple liver conditions, including MASLD, ALD, AILD, liver cirrhosis, and HCC. Therefore, targeted restoration and improvement of the gut immune barrier to restore gut–liver axis homeostasis has emerged as a novel, highly promising strategy for liver disease treatment (Figure 3).

### 4.1. Preclinical-Stage Strategy

#### 4.1.1. Universal Strategies (Applicable to Multiple Liver Diseases)

Currently, the primary universal strategies include broad-spectrum therapies such as prebiotics, fecal microbiota transplantation (FMT), intestinal cleansing, and nutritional support. These approaches aim to modulate the intestinal immune microenvironment by regulating the balance of the gut microbiota, thereby repairing the intestinal barrier and inhibiting common downstream inflammatory pathways, ultimately treating liver diseases via the gut–liver axis [5,76,77]. While these therapies may offer broad-spectrum efficacy and relatively low side effects, their effectiveness varies significantly between different batches. Moreover, they lack precision, specificity, and targeting capability.

Currently, there are few reports on alleviating liver disease by targeting cells and molecular pathways in the gut immune microenvironment. Blocking core gut–liver axis signaling pathways—including cells such as macrophages and T cells, pathways like LPS-TLR4, and antibodies like pIgA—holds theoretical therapeutic value across all relevant stages of liver disease. We hope that future research will provide new hope for treating liver disease through the modulation of the gut immune microenvironment.

#### 4.1.2. Targeted Strategies for Specific Liver Diseases

As previously mentioned, one of the inflammatory pathways in MASLD is mediated by KCs, which can be activated by TLRs. The gut microbiota can exacerbate host inflammation through various mechanisms. Previous studies have demonstrated that the absence of specific TLRs or the use of innate immune adaptors such as MyD88 exerts a protective effect against HFD-induced obesity [78,79]. Targeting the intestinal immune microenvironment provides a novel perspective for MASLD treatment. Multi-level intervention in the “intestinal barrier-microbiota-immunity” axis makes it possible to disrupt the vicious cycle of MASLD progression.

Recent studies indicate that in ALD, inactivated postbiotics can activate intestinal innate immunity through the Gut DCs-ILC3 axis, upregulate IL-22 production, promote intestinal epithelial regeneration, strengthen the gut barrier at the upstream level, reduce bacterial translocation, and subsequently exert reparative effects on the liver via the gut–liver axis [46]. With deeper understanding of gut immunity mechanisms in ALD, more precisely targeted therapies are expected to emerge in the future, offering new management strategies for this refractory liver disease.

Intestinal inflammation increases the number of gut-homing lymphocytes (expressing α4β7/CCR9), while their ligands (MAdCAM-1/CCL25) are abnormally expressed in the liver, causing these cells to launch a misdirected attack on the hepatobiliary system, particularly in PSC [59]. Consequently, the targeted drug vedolizumab, an anti-α4β7 integrin antibody, may offer a novel therapeutic option for AILD [53,59,80].

Regulatory T cell induction, namely, low-dose IL-2 therapy, can suppress excessive hepatic inflammation and fibrotic responses by enhancing gut immune regulatory function [81]. IL-22 Fc fusion protein and S-adenosylmethionine (SAMe) also enhance gut immune regulatory function: IL-22 promotes epithelial repair, while SAMe provides methyl groups to support hepatic anti-inflammatory and antioxidant functions [15].

Multi-omics analysis of patients with HBV-associated HCC has revealed a strong correlation between specific gut microbiota features and the status of the tumor immune microenvironment (e.g., CD8^+^ T-cell infiltration) as well as patient prognosis. This provides direct evidence for a precision combination strategy aimed at improving the immune microenvironment by modulating the microbiota, thereby enhancing the efficacy of PD-1 inhibitors [82]. Another study indicated that blocking the CCL2/CCR2 axis can inhibit the recruitment of tumor-promoting M2-type macrophages into liver tumor tissue. Given that intestinal inflammation is a major source of circulating CCL2, therapies targeting the intestinal barrier (e.g., reducing LPS entry into the bloodstream) may indirectly suppress this pathway and exert synergistic anti-tumor effects with CCR2 antagonists [83]. Anti-cancer therapies targeting the gut-liver axis may emerge as a novel strategy to enhance the efficacy of immunotherapies for liver cancer, such as PD-1 inhibitors, when used in combination. By improving intestinal immune and barrier functions, it is possible to remodel the hepatic immune microenvironment, thereby converting “cold” tumors into “hot” tumors.

Gut–liver axis-targeted therapy centered on improving the gut immune barrier represents a profound shift in the liver disease management paradigm–from solely treating the liver to treating both the liver and gut. Universal strategies focus on restoring the barrier’s infrastructure and function, providing a therapeutic starting point applicable to various liver diseases. Conversely, targeted strategies for specific liver diseases reflect a personalized therapeutic approach based on a precision-based understanding of pathological mechanisms.

### 4.2. Clinical Stage Strategy

Currently, drugs that alleviate liver diseases by modulating the gut–liver axis immune microenvironment, with evidence from human clinical trials, are primarily concentrated in the fields of MASH and AILDs.

#### 4.2.1. Obeticholic Acid: Farnesoid X Receptor Agonist

Intestinal FXR activation exerts potent immunomodulatory effects with anti-inflammatory and anti-fibrotic properties: it inhibits the activation of Kupffer and hepatic stellate cells and reduces the production of pro-inflammatory cytokines. The REGENERATE study (Phase III): After 18 months of treatment, the proportion of patients with MASH and stage 2–3 fibrosis whose liver fibrosis improved by ≥1 stage without the worsening of MASH was significantly higher in the obeticholic acid 25 mg dose group compared to the vehicle group (23.1% vs. 11.9%), making it the first MASH drug to meet the primary endpoint in a Phase III trial [84]. Application in PBC: Obeticholic acid has been approved by the FDA for patients with PBC who inadequately respond to ursodeoxycholic acid, with effects including improvement in cholestasis and alleviation of pruritus [85].

#### 4.2.2. Cenicriviroc: Chemokine Receptor 2/5 Dual Antagonist

Intestinal CCL2/CCL5 are key chemoattractants that recruit inflammatory monocytes and T cells to the liver via receptors CCR2 and CCR5, respectively. Specifically inhibiting the migration and retention of pro-inflammatory, pro-fibrotic monocytes/macrophages to the liver thus ameliorates hepatic inflammation and fibrosis. The CENTAUR study (Phase IIb): After one year of treatment, the proportion of patients with MASH and stage 2–3 fibrosis who exhibited “improvement in fibrosis by ≥1 stage without worsening of MASH” was significantly higher in the CVC group compared to the vehicle group (16% vs. 10%) [86].

#### 4.2.3. Lanifibranor: Peroxisome Proliferator-Activated Receptor α/δ Dual Agonist

PPAR-α regulates fatty acid oxidation, while PPAR-δ regulates fatty acid metabolism and possesses anti-inflammatory effects. By activating both receptors, lanifibranor comprehensively modulates lipid metabolism in the liver and systemically, and directly inhibits hepatic inflammation and fibrosis. The NATIVE study (Phase IIb): After 24 weeks of treatment with lanifibranor (1200 mg/day) in patients with MASH, the proportion of patients who achieved the primary endpoint of “MASH resolution without worsening of fibrosis” was significantly higher compared to the vehicle group (55% vs. 33%) [87]. The drug also showed a positive trend in improving fibrosis.

Targeting the gut–liver axis immune microenvironment has become a prominent avenue in liver disease drug development. The aforementioned clinical-stage drugs not only validate the therapeutic potential of several key targets but also provide valuable human data for understanding gut–liver immune crosstalk in liver diseases, driving the field toward a more precise and effective era of treatment.

## 5. Conclusions and Discussion

The gut immune microenvironment engages in intricate information exchange with the liver via the gut–liver axis. This dysregulated crosstalk serves as a critical driver in the formation and progression of pathological damage to the liver. The gut immune microenvironment is not an independent layer of the gut barrier; rather, it permeates and regulates all other barrier functions, serving as the core coordinator. The homeostasis of the immune microenvironment serves as a cornerstone for maintaining overall barrier integrity and is the root cause of various intestinal and extraintestinal diseases (such as inflammatory bowel disease and liver disease). Disruption of the immune microenvironment in the gut–liver axis drives progression across the full disease spectrum–from steatosis to hepatitis, fibrosis, and even liver cancer–through the continuous influx of immune-stimulatory signals that overwhelm the liver’s intrinsic immune regulatory mechanisms. The disruption of innate immunity mediated by macrophages, the amplification of inflammatory factors and key cellular signaling pathways, the activation of adaptive immune T cells, and the systemic impact of liver-derived inflammatory factors collectively create a disordered immune microenvironment. This impairs the gut barrier, exacerbates liver disease via the gut–liver axis, and damages the hepatic immune microenvironment. Consequently, the gut suffers further impacts, establishing a vicious cycle. Current therapeutic strategies based on modulating the gut–liver axis microenvironment remain limited, yet studies have demonstrated that suppressing gut immune cells, cytokines, and signaling pathways can help delay liver disease progression.

### 5.1. Gut Microbiome Multi-Omics

Beyond therapeutic innovation, the gut–liver axis paradigm is also revolutionizing the diagnostic landscape for chronic liver diseases. A significant frontier lies in the application of high-throughput multi-omics technologies, particularly metagenomic and metabolomic profiling of the gut microbiome. Unlike traditional culture-based methods, shotgun metagenomic sequencing allows for the comprehensive characterization of the gut microbial community’s taxonomic composition and functional potential (e.g., genes involved in bile acid metabolism or LPS biosynthesis). Complementarily, metabolomics directly measures the repertoire of microbial-derived metabolites (such as secondary bile acids, short-chain fatty acids, and trimethylamine N-oxide) in the stool or blood, providing a functional readout of host–microbe crosstalk [88,89,90].

Emerging studies have identified distinct microbial signatures and metabolite profiles associated with MASLD severity (e.g., increased Escherichia and decreased Faecalibacterium), progression to MASH, and advanced fibrosis [13,19]. These microbial biomarkers hold promise for developing non-invasive diagnostic tools to stratify disease risk, differentiate steatosis from steatohepatitis, and monitor treatment response. However, translating these findings into clinically validated assays requires the standardization of protocols, validation in large, diverse cohorts, and demonstration of cost-effectiveness. Future integration of microbial multi-omics data with host clinical and genetic variables may pave the way for precision diagnostics, enabling earlier intervention and tailored management strategies based on an individual’s gut ecosystem status.

### 5.2. From the Preclinical to Clinical Treatment Stage: Opportunities and Challenges of Targeted Therapies for the Intestinal Immune Microenvironment

Although targeting the intestinal immune microenvironment offers a promising new paradigm for the treatment of liver diseases, its translation into clinical success remains a significant challenge. This section aims to critically compare the main strategies, analyze their inherent limitations and the reasons for clinical failures, and explore how these obstacles might be overcome through innovative design.

Direct Immune-Targeting Therapies. These approaches target specific immune cells (e.g., Tregs, Th17), cytokines (e.g., IL-17, IL-23), or checkpoints (e.g., local intestinal PD-1/PD-L1). Their advantages include direct action and high potency. However, they also have several disadvantages: systemic administration may disrupt systemic immune homeostasis; significant inter-patient heterogeneity in immune phenotypes makes single-target strategies difficult to generalize universally; blockade of a single pathway may lead to compensatory activation of others; and the therapeutic time window is narrow—intervention may be ineffective or even harmful if administered too late (e.g., after cirrhosis is established). Current research indicates that anti-IL-17/IL-23 antibodies are effective in IBD, but have largely failed to meet primary endpoints in clinical trials for MASLD [46,78,79].

Microbiota-Based Therapies. These approaches aim to holistically reshape the gut microbiota composition (e.g., FMT, probiotics) or its metabolic output (e.g., postbiotics, prebiotics). Their advantages include addressing the root causes and offering pleiotropic effects, potentially improving barrier function, metabolism, and immunity simultaneously, with a relatively favorable safety profile. Their disadvantages encompass unclear causality between microbial shifts and outcomes; vast inter-individual variability in responses; challenges in quality control due to product complexity; and transient effects. Clinical outcomes for specific probiotic combinations have been inconsistent, and FMT lacks large-scale randomized controlled trials (RCTs) [76,77]. Intestinal Barrier Repair Strategies. These strategies focus on enhancing tight junctions (e.g., via lactobacillus-derived products), promoting mucus production, and providing nutrients for enterocytes (e.g., glutamine) [5,76,77]. Their advantages include the potential to prevent or delay disease onset and a low side-effect profile. Their disadvantages are that merely repairing the barrier may be insufficient during active inflammatory flares; they cannot target specific intestinal segments; and the lack of specific biomarkers makes it difficult to identify patients most likely to benefit from barrier repair therapies.

Given the above discussion, the clinical translation of targeting the gut–liver axis in extraintestinal diseases faces considerable difficulties. The complexity of the gut–liver axis system, the fundamental differences between animal models and humans, the lack of patient biomarkers, and the challenge of achieving high local drug concentrations with sustained action in the intestine while minimizing systemic exposure and associated risks are pressing issues that require resolution.

Future therapies for liver diseases targeting the immunomodulation of the gut–liver axis are primarily focused on the following aspects. First, combination strategies, such as integrating immune-targeting agents with probiotics or FMT, aim to achieve synergistic effects [91,92]. Second, precision approaches involve analyzing the intestinal immune profiles of patients to enable personalized interventions. Third, cutting-edge developments, including engineered probiotics, specific bacteriophages, or nanocarriers, are being explored to achieve more precise regulation of the gut microenvironment and immune signaling [48,93,94,95]. Fourth is precision medicine. As mentioned earlier, in the future, clinical trials must integrate multi-dimensional data such as the metagenome of the intestinal microbiota, the map of immune cells, and metabolomics to discover biomarkers that predict therapeutic efficacy and achieve patient stratification. However, challenges remain: the long-term safety and efficacy of many interventions require validation through large-scale clinical trials, and the optimal timing and combination strategies for different stages of liver disease need to be clarified. Future research should further elucidate the molecular mechanisms underlying the crosstalk between specific intestinal immune cell subsets and the liver. In summary, the notion that safeguarding the intestinal immune microenvironment is integral to preserving liver health is currently guiding—and will continue to drive—innovation in the therapeutics for liver diseases.

## Figures and Tables

**Table 1:** Key gut immune regulatory targets and pathways in MASLD.

Liver Disease	Immune Cells/Factors	Localization	Primary Functions	Key Pathways	References
MASH	Th17 cells	Murine intestinal lamina propria	Drive of intestinal inflammation and barrier breakdown. These cells expand under dysbiotic conditions, exacerbating local gut inflammation and promoting barrier dysfunction.	RORγt-mediated differentiation; IL-17A and IL-22 secretion.	[20]
MASLD	IL-33	Murine intestinal mucus barrier	Drive of intestinal inflammation and mucus barrier disruption.	Promotion of the synthesis of gut microbiota-derived trimethylamine N-oxide via gut-derived IL-33, through dual regulation of hypoxia-inducible factor-1α and fueling of MASLD progression.	[21]
MASH	CXCR6^+^ CD8^+^ T cells	Mouse liver	The “executors” attack hepatocytes. Activated by gut-derived antigens, these key effector cells drive the transition from simple steatosis to MASH by directly inducing hepatocyte ballooning and death through IFN-γ production.	Key mechanisms: Gut antigen-dependent activation; CXCR6^−^ mediated intrahepatic retention; IFN-γ secretion inducing hepatocyte death.	[22]
MASH/Hepatic Fibrosis	NLRP3 Inflammasome-activated macrophages	Murine liver (Kupffer and monocyte-derived cells)	An “amplifier” of liver inflammation and a “driver” of fibrosis. Upon activation by signals such as gut-derived LPS, they produce substantial levels of IL-1β and IL-18, directly exacerbating the hepatocyte inflammation–injury cycle and potently activating hepatic stellate cells to promote fibrosis.	TLR4/NLRP3/IL-1β signaling axis.	[23]
MASLD/MASH	Treg	Murine intestinal lamina propria & liver	The failing brake of immune homeostasis. In disease states, the number of Treg cells in the gut and liver may decrease or their function may be compromised, which makes them unable to effectively suppress the overactivation of effector cells such as Th17 and CD8^+^ T cells, leading to an imbalance in immunosuppression.	Secretion of IL-10 and TGF-β; cell contact-dependent suppression.	[24]
MASH	Intestinal macrophages (CX3CR1^+^)	Murine intestinal lamina propria	The supplier of barrier support and the regulator of inflammation. Specific subsets maintain intestinal vascular barrier integrity through the secretion of WNT ligands. Its dysfunction weakens the secondary line of defense, while its phenotypic shift toward pro-inflammatory (M1) exacerbates local inflammation.	WNT/β-catenin pathway (maintains barrier); TLR signaling (promotes pro-inflammatory shift).	[9]
MASLD progression	Hepatocytes	Mouse liver	A new contributor to inflammation. Upon direct uptake of gut-derived metabolites (e.g., TMAO), hepatocytes can autonomously activate the NLRP3 inflammasome, undergo pyroptosis, and release IL-1β, amplifying intrahepatic inflammation from a non-immune cell perspective.	Direct activation of hepatocyte NLRP3 by the metabolite TMAO.	[25]
MASLD	CD8^+^ T cells	Human jejunum	Gut barrier breakdown. In obese patients, the number of CD8^+^ T cells in the jejunum increases. Certain soluble factors secreted by these cells significantly inhibit phospho-Ser473-Akt activity in intestinal epithelial cells, thereby impairing insulin sensitivity in small intestinal epithelial cells.	CD8^+^ T cell-induced inhibition of intestinal epithelial cell PKB.	[26]
MASLD	CD4^+^ and CD8^+^ T cells	Human duodenum	Gut barrier compromised. In patients with MASLD and metabolic dysregulation, the duodenum exhibits significantly reduced CD4^+^ and CD8^+^ T lymphocytes along with decreased ZO-1 expression, leading to impairment of the intestinal mechanical barrier, thus further causing selective loss of CD4^+^ T lymphocytes in the human liver	Significant decrease in expression of CD4^+^ and CD8^+^ T lymphocytes along with ZO-1.	[27,28]

**Table 2:** Key gut immune regulatory targets and pathways in ALD.

Liver Disease	Immune Cells/ Factors	Localization	Primary Functions	Key Pathways	References
ALD	IL-17-producing γδ T cells	Murine intestinal lamina propria/intraepithelial	These cells are initiators that disrupt the gut barrier. Their aberrant expansion and activation directly impair intestinal epithelial tight junctions, leading to increased gut permeability, representing an early event in leaky gut associated with alcoholic liver disease.	These cells secrete cytokines such as IL-17, disrupting the expression of interepithelial junction proteins.	[16]
ALD	Gut-derived Th17 cells	Gut → Migration to liver	1. These cells are induced in the gut by alcohol/dysbiosis. 2. They migrate to the liver and secrete IL-17A. 3. They recruit neutrophils, driving liver inflammation and injury.	CCL20-CCR6 axis: Gut-derived CCL20 recruits CCR6-expressing Th17 cells to the liver. Bacterial products (e.g., LPS) act as adjuvants to promote Th17 differentiation.	[38]
ALD	Liver-resident macrophages (Kupffer cells)	Liver (liver sinusoids)	1. These cells receive PAMPs/DAMPs (e.g., LPS, bacterial DNA) from the gut. 2. These result in excessive activation of pathways such as TLR4/MyD88. 3. These cells generate large amounts of TNF-α, IL-1β, and reactive oxygen species, inducing hepatocyte steatosis, inflammation, and apoptosis.	TLR4/MyD88/NF-κB pathway: This is the core inflammatory pathway activated by LPS. NLRP3 inflammasome: it is activated by various gut-derived signals, releasing IL-1β.	[39]
ALD	Liver-resident immune cells	Liver	These cells significantly repair the damaged gut epithelial barrier by enhancing the expression of intestinal tight junction proteins and suppressing the production of gut-derived lipotoxins.	AGE prevents alcoholic liver disease by modulating the gut–liver axis and suppressing the TLR 4/NF-kB/MLKL-mediated necroptosis pathway.	[40]
ALD/LC	Bone marrow-derived monocytes/macrophages	Bone marrow → circulation → liver Infiltration	1. Intestinal leakage leads to systemic inflammation. 2. Monocytes are recruited to the liver and differentiate into pro-inflammatory (M1-type) macrophages. 3. This exacerbates liver inflammation and promotes hepatic stellate cell activation, driving fibrosis.	CCL2-CCR2 axis: Damaged hepatocytes and Kupffer cells produce CCL2, recruiting CCR2-expressing monocytes to the liver.	[41]
ALD	Gut lamina propria B cells/IgA^+^ plasma cells	Intestinal lamina propria	1. Functional impairment: Alcohol inhibits gut B cells from secreting protective IgA. 2. Functional alteration: Dysbiosis causes B cells to produce abnormal antibodies against commensal bacteria. 3. This leads to decreased mucosal immune defenses, increased bacterial overgrowth, and enhanced translocation.	Retinoic acid metabolic pathway: Alcohol disrupts retinoic acid (a vitamin A metabolite) synthesis, which serves as a critical signal for intestinal B cells to differentiate into plasma cells and produce IgA.	[42]
ALD	Intestinal intraepithelial lymphocytes	Intestinal epithelial layer	1. Hypercytotoxicity: Under alcohol influence, IELs (particularly γδ T cells) excessively produce cytokines and granzymes. 2. These cells directly attack and damage intestinal epithelial cells, compromising gut barrier integrity.	Microbiota-dependent activation: Microbial products presented by epithelial cells aberrantly activate IELs. IL-15 pathway: IL-15 produced by epithelial cells serves as a critical factor for IEL maintenance and activation.	[43]

**Table 3:** Key gut immune regulatory targets and pathways in AILD.

Liver Disease	Immune Cells/ Factors	Localization	Primary Functions	Key Pathways	References
PBC	Gut-homing IgA^+^ plasma cells	Gut → Migration to liver	1. These cells are activated by antigens (e.g., commensal bacteria) in the intestinal mucosa and differentiate into IgA^+^ plasma cells. 2. These cells abnormally migrate to the portal areas of the liver. 3. They produce pIgA antibodies targeting gut commensals (e.g., Enterococcus faecalis), forming immune complexes that deposit around bile ducts and cause bystander injury to bile ducts through complement pathway activation.	Gut microbiota–mucosal immunity–liver homing: Specific gut microbiota drive IgA-producing B cells, which subsequently undergo aberrant homing to the liver and produce antibodies. pIgR-mediated transport: Bile duct epithelial cells express polymeric immunoglobulin receptor (pIgR), potentially involved in transporting aberrant IgA and mediating local immune responses.	[53,54]
AIH	Gut-derived Th17 cells	Gut → Migration to liver	1. Intestinal barrier breakdown (Leaky gut) leads to translocation of bacteria/food antigens. 2. Th17 cells migrate to the liver, secrete IL-17 and IL-22, recruit neutrophils, and promote hepatocyte damage and portal inflammation.	IL-6/TGF-β/IL-23 pathway: This is a key cytokine pathway driving Th17 differentiation, which can be activated by gut dendritic cells upon stimulation by microbiota. CCR6-CCL20 Axis: Liver-damaged cells express CCL20, recruiting CCR6-expressing Th17 cells.	[55]
PBC	Microbiota-mimicking activated T/B cells	Systemic circulation, locally activated in the liver	1. Proteins expressed by certain gut microbiota bacteria exhibit structural similarities to human mitochondrial antigens (e.g., PDC-E2). 2. Due to barrier defects, the gut immune system encounters these bacteria and generates cross-reactive antibodies and T cells.	Molecular mimicry: Cross-reactivity between gut microbiota antigens and self-antigens constitutes the core mechanism breaking immune tolerance. Apoptotic cell antigen exposure: Damaged bile duct epithelial cells abnormally expose PDC-E2 antigens, becoming targets of autoimmune attack.	[56,57]
AIH	Liver sinusoidal endothelial cells (LSECs)	Liver (liver sinusoids)	1. LSECs serve as the first immune barrier encountered by gut-derived antigens (including bacterial and food antigens) upon entering the liver. 2. During gut–liver axis dysregulation, LSECs abnormally overexpress vascular adhesion protein-1 (VAP-1) and mucosal addressin cell adhesion molecule-1 (MAdCAM-1).	VAP-1-mediated lymphocyte recruitment: VAP-1 is a key molecule on LSECs that induces lymphocyte rolling, adhesion, and extravasation, with its expression upregulated in PSC and AIH. Antigen cross-presentation: LSECs can cross-present gut-derived antigens, potentially activating or reactivating autoreactive T cells within the liver.	[58]
PSC	Gut-derived CCR9^+^ memory T cells	Gut → Migration to liver/bile duct	1. These cells are activated in the inflamed gut in IBD (especially UC). 2. They express gut-homing receptors CCR9 and α4β7.	CCL25-CCR9 axis and MAdCAM-1/α4β7 integrin axis: The liver’s abnormal expression of gut-homing ligands constitutes the core of the gut–liver lymphocyte homing theory, directly linking IBD with PSC.	[59]

**Table 4:** Key gut immune regulatory targets and pathways in LC.

Liver Disease	Immune Cells/Factors	Localization	Primary Functions	Key Pathways	References
LC	Gut-derived LPS-activated hepatic macrophages	Liver	1. Detect translocated LPS from ‘leaky gut’ and transform into a pro-fibrotic phenotype. 2. By secreting cytokines such as TGF-β1 and PDGF, they directly activate hepatic stellate cells, inducing their transformation into collagen-producing myofibroblasts. 3. Creating a sustained inflammatory microenvironment that perpetuates the fibrotic process.	TLR4-MyD88-NF-κB pathway: LPS activates this pathway, inducing expression of pro-inflammatory and pro-fibrogenic factors. CCL2-CCR2 axis: Activation of this pathway drives further monocyte infiltration, replenishing the fibrosis-associated macrophage pool.	[61]
Biliary atresia/Cholestatic hepatic fibrosis	α4β7^+^ T lymphocytes homing to the gut	Gut → Migration to liver	1. Intestinal inflammation leads to an increase in T cells expressing the gut-homing receptor α4β7. 2. Under inflammatory conditions, bile duct epithelial cells in the liver abnormally express mucosal addressin cell adhesion molecule-1 (MAdCAM-1), acting as a misleading signal. 3. α4β7^+^ T cells are aberrantly recruited to the liver, attacking bile ducts and exacerbating biliary injury and fibrosis in the portal area.	MAdCAM-1/α4β7 integrin axis: This is the classic gut–liver lymphocyte homing pathway. Aberrant expression of MAdCAM-1 in the liver represents a pivotal mechanism linking gut immunity to hepatic pathology.	[62]
LC	Bone marrow-derived Ly6C monocytes	Bone Marrow → Circulation → Liver Infiltration	1. Gut dysbiosis and barrier breakdown induce systemic chronic low-grade inflammation. 2. This drives heightened production of pro-inflammatory Ly6C monocytes in the bone marrow. 3. Recruited to the injured liver, these monocytes differentiate into pro-fibrotic macrophages and serve as a primary cellular source for intrahepatic collagen deposition.	NLRP3 inflammasome/IL-1β axis: Gut-derived substances (e.g., cholesterol crystals, LPS) activate the NLRP3 inflammasome in the liver and bone marrow, producing IL-1β, which drives myeloid cell proliferation and activation. CCR2-CCL2 axis: This is a critical recruitment pathway.	[63]
LC	Impaired regulatory T cell function	Intestinal lamina propria and liver	1. In the gut, alcohol and toxins impair Treg function, weakening immune tolerance to gut microbiota and exacerbating inflammation and leaky gut. 2. In the liver, the fibrotic microenvironment (high TGF-β) should promote Treg differentiation to suppress immunity. However, persistent gut-derived stimuli cause pro-inflammatory responses to predominate, resulting in relatively insufficient Treg inhibitory function that fails to effectively control HSC activation and macrophage inflammation.	The differentiation and function of gut Tregs are heavily reliant on retinoic acid (vitamin A metabolites) produced by gut cells. This pathway is often impaired in liver disease. Weakened IL-10 signaling: This involves reduced production of or responsiveness to IL-10, a key anti-inflammatory factor secreted by Tregs.	[42,64]

**Table 5:** Key gut immune regulatory targets and pathways in HCC.

Liver Disease	Immune Cells/Factors	Localization	Primary Functions	Key Pathways	References
HCC	Bone marrow-derived suppressor cells	Gut (influences production) → Liver (aggregation)	1. Gut dysbiosis (e.g., overgrowth of Helicobacter hepaticus) and ‘leaky gut’ induce systemic chronic inflammation. 2. This drives the massive production of MDSCs in the bone marrow. 3. MDSCs recruited to the liver cancer microenvironment strongly suppress the anti-tumor function of CD8^+^ T cells and NK cells, promoting tumor growth and metastasis.	TLR4/MyD88 signaling: Gut-derived LPS promotes differentiation of myeloid precursors into MDSCs through TLR4 activation. STAT3 Pathway: Persistent inflammatory signals activate STAT3, serving as the core mechanism for MDSC expansion and functional maintenance. CCL2-CCR2 axis: Recruits MDSCs to tumor sites.	[71]
HCC	Immunosuppressive tumor-associated macrophages	Liver (tumor microenvironment)	1. Gut-derived substances (e.g., LPS, deoxycholic acid) polarize hepatic macrophages toward M2-like/immunosuppressive TAMs. 2. TAMs secrete IL-10, TGF-β, etc., suppressing effector T cell function. 3. TAMs highly express checkpoint molecules such as PD-L1, directly contributing to T cell exhaustion. 4. TAMs secrete pro-angiogenic factors to promote tumor angiogenesis.	DCA-FXR axis: Secondary bile acid deoxycholic acid produced by gut microbiota promotes M2-type TAM polarization through farnesoid X receptor signaling. LPS-TLR4-NF-κB pathway: This pathway drives pro-tumor inflammation and immunosuppressive phenotypes.	[72]
HCC	Exhausted/dysfunctional CD8^+^ T cells	Liver (tumor microenvironment)	1. The immunosuppressive microenvironment created by the aforementioned MDSCs and TAMs directly leads to functional exhaustion of infiltrating tumor-specific CD8^+^ T cells. 2. These T cells highly express inhibitory receptors such as PD-1, TIM-3, and CTLA-4, lose their proliferative and cytotoxic capabilities, and fail to eliminate tumor cells.	PD-1/PD-L1 pathway: Both TAMs and tumor cells highly express PD-L1, which binds to PD-1 on T cells, delivering inhibitory signals. Metabolic competition: In the tumor microenvironment, MDSCs and tumor cells competitively consume nutrients such as arginine and tryptophan, leading to metabolic exhaustion of T cells.	[73]
HCC	Regulatory T cells	Gut (influences production) → Liver (aggregation)	1. Specific gut microbiota and their metabolites (e.g., short-chain fatty acids–SCFAs) promote the generation and function of regulatory T cells in both the gut and peripheral tissues. 2. In the context of liver cancer, excessive Tregs are recruited to tumor sites. 3. High levels of Tregs in the tumor microenvironment further suppress the activity of effector T cells and NK cells, facilitating tumor immune escape.	SCFAs-GPR43/HDAC inhibition axis: SCFAs (e.g., butyrate) produced by the gut microbiota through dietary fiber fermentation promote Treg differentiation via G protein-coupled receptors or histone deacetylase inhibition. TGF-β signaling: Both tumor microenvironment and gut-derived signals can induce Tregs through TGF-β.	[74]
HCC	Liver natural killer cells	Liver (tumor microenvironment)	1. NK cells serve as the first responders of anti-tumor immunity in the liver. 2. Gut dysbiosis and associated alterations in bile acid metabolism directly inhibit the cytotoxic activity and IFN-γ production capacity of NK cells. 3. This leads to the impairment of immune surveillance function, enabling the escape and growth of early-stage tumor cells.	Bile acid–NKG2D pathway: Certain gut microbiota-dependent bile acids (e.g., lithocholic acid) can downregulate the expression of the activating receptor NKG2D on NK cells, thereby weakening their recognition and killing of tumor cells.	[71]

## Data Availability

The original contributions presented in this study are included in the article. Further inquiries can be directed to the corresponding author.

## Abbreviations

The following abbreviations are used in this manuscript:

LC	Liver Cirrhosis
HCC	Hepatocellular Carcinoma
MASLD	Metabolic Dysfunction-Associated Steatotic Liver Disease
ALD	Alcoholic Liver Disease
AILD	Autoimmune Liver Disease
PBC	Primary Biliary Cholangitis
AIH	Autoimmune Hepatitis
PSC	Primary Sclerosing Cholangitis
GALT	Gut-associated Lymphoid Tissue
GVB	Gut Vascular Barrier
LPS	Lipopolysaccharide
VRE	Vancomycin-Resistant Enterococcus
SFB	Segmented Filamentous Bacteria
IECs	Intestinal Epithelial Cells
AMPs	Antimicrobial Peptides
sIgA	secretory Immunoglobulin A
Treg	Regulatory T Cell
DCs	Dendritic Cells
PAMPs	Pathogen-associated Molecular Patterns
DAMPs	Damage-associated Molecular Patterns
TNF-α	Tumor Necrosis Factor-α
MAT	Mesenteric Adipose Tissue
IL	Interleukin
MDSCs	Myeloid-derived Suppressor Cells
MASH	Metabolic Dysfunction-Associated Steatotic Hepatitis
CX3CR1	CX3C chemokine receptor 1
NOD	Nucleotide-binding Oligomerization Domain
BT	Bacterial Translocation
BATF3	Basic leucine zipper ATF-like Transcription Factor 3
HFD	High-fat Diet
MLNs	Mesenteric Lymph Nodes
HSCs	Hepatic Stellate Cells
GAPs	Goblet cell associated Antigen Passages
EVs	Extracellular Vesicles
ACLF	Acute-on-Chronic Liver Failure
TAMs	Tumor-associated Macrophages
FMT	Fecal Microbiota Transplantation
SAMe	S-adenosylmethionine
FXR	Farnesoid X Receptor
RCTs	Randomized Controlled Trials
